# A Wireless Passive Sensing System for Displacement/Strain Measurement in Reinforced Concrete Members

**DOI:** 10.3390/s16040496

**Published:** 2016-04-08

**Authors:** Burak Ozbey, Vakur B. Erturk, Hilmi Volkan Demir, Ayhan Altintas, Ozgur Kurc

**Affiliations:** 1 Department of Electrical and Electronics Engineering, Bilkent University, 06800, Ankara, Turkey; vakur@ee.bilkent.edu.tr (V.B.E.); volkan@bilkent.edu.tr (H.V.D.); altintas@ee.bilkent.edu.tr (A.A.); 2 School of Electrical and Electronic Engineering, Nanyang Technological University, Singapore; 3 Department of Civil Engineering, Middle East Technical University, Ankara TR-06800, Turkey; kurc@metu.edu.tr

**Keywords:** wireless passive sensors, metamaterial based sensors, structural health monitoring, simply supported beam experiment, elastic-plastic deformation of steel, strain measurement, displacement measurement

## Abstract

In this study, we show a wireless passive sensing system embedded in a reinforced concrete member successfully being employed for the measurement of relative displacement and strain in a simply supported beam experiment. The system utilizes electromagnetic coupling between the transceiver antenna located outside the beam, and the sensing probes placed on the reinforcing bar (rebar) surface inside the beam. The probes were designed in the form of a nested split-ring resonator, a metamaterial-based structure chosen for its compact size and high sensitivity/resolution, which is at µm/microstrains level. Experiments were performed in both the elastic and plastic deformation cases of steel rebars, and the sensing system was demonstrated to acquire telemetric data in both cases. The wireless measurement results from multiple probes are compared with the data obtained from the strain gages, and an excellent agreement is observed. A discrete time measurement where the system records data at different force levels is also shown. Practical issues regarding the placement of the sensors and accurate recording of data are discussed. The proposed sensing technology is demonstrated to be a good candidate for wireless structural health monitoring (SHM) of reinforced concrete members by its high sensitivity and wide dynamic range.

## 1. Introduction

Readily-employed technologies to monitor relative displacement and strain in a reinforced concrete member with sensors such as strain gages are generally wired. In other words, the sensor must be connected to a data acquisition system at all times. This requirement makes them unpractical to be used in an actual structure and limits their usage in structural health monitoring (SHM) [1]. It is, thus, important that the utilized measurement system enables wireless exchange of data, and the sensing device embedded inside the concrete does not need electrical energy to function (passive structure). Hence, a wireless, passive displacement/strain measurement system for reinforced concrete members enables the monitoring of deformation of such members in a much practical manner. In the literature, different schemes have been proposed with the purpose of sensing the strain and/or displacement as well as other types of damage indices such as corrosion, wirelessly and by a passive sensor geometry. Among these, radio-frequency identification (RFID)-based methods [2,3,4,5] have become increasingly popular. Especially, passive RFID is an emerging technique where a passive tag transfers the information about the sensed quantity, which can include, for example, temperature, humidity and density of chemical compounds as well as strain and relative displacement, via impedance change to the transmitter. The transmitter in turn sends the digitalized signal wirelessly to the receiver. Subsequently, the signal collected by the receiver is further processed to obtain the change of the sensed quantity in terms of radar cross section or turn-on power of the RFID chip. A semi-passive system is possible by converting the interrogating radio frequency (RF) signal to direct current so that it is used to feed the transmitter, making the passive-RFID quite appealing. Nevertheless, RFID-based displacement sensors can only offer a resolution in the order of millimeters [3,4]. This constitutes a major problem when it is considered that generally micrometer-scale changes are observed and required to be measured in SHM applications [6]. Yi *et al.* have demonstrated a resolution as low as 20 microstrains by an RFID-based strain sensor at a laboratory environment, but the operation was limited to free-space [7].

Another frequently used strain sensing technology is the surface acoustic wave (SAW) [8,9,10]. SAW-based sensors monitor strain by detecting the change in acoustic wave velocity due to deformation. Excess amount of losses and difficulty in creating coupling between the acoustic waves and the sensed quantity are, however, serious drawbacks of these systems [11,12]. Previously, antennas that are utilized as sensors for detecting the sensed quantity were reported using the measured changes in the radiation pattern of the antenna stemming from deformation [7,13,14,15,16]. Other wireless and passive strain sensors include magnetostrictive sensors, which can change their shapes with changing magnetic fields [17,18]; microfluidic sensors in which the flow of a liquid in a very thin channel is monitored ultrasonically [19,20]; smart skin sensors [21]; flexible LC circuit-based designs [22,23,24]; and RF cavity transducers where the cavity resonance frequency is a function of strain [25]. On the other hand, none of these methods have been able to find a widespread area of use due to major limitations including difficulty of implementation, complexity of design, and/or limited sensitivity and resolution. Other wireless techniques including piezoelectric transducers [26,27], frequency doubling sensors [28], and microelectromechanical systems (MEMS) accelerators [29], were also shown for different SHM applications in recent years.

Metamaterials are artificially engineered structures that are manufactured by assembling at least two types of materials (or more) with sufficiently different intrinsic properties in a repetitive manner such that the overall structure exhibits an electromagnetic behavior which is different than that of both the base materials and most probably from any other material that can naturally be found, such as negative refraction index [30], reversed Doppler effect [31], and cloaking [32]. In recent years, metamaterials and metamaterial-based structures have also been employed for the purpose of sensing, including strain and displacement sensors [33,34,35,36,37,38]. More recently, a metamaterial-based structure, which is a type of a nested split-ring resonator (NSRR) was proposed by Melik *et al.* [39]. This structure, called as the comb-like NSRR (or just NSRR), was shown to be highly convenient for sensitive strain measurements with its high field localization. The original structure was previously modified to serve primarily as a displacement sensor by our research team [40], where it was demonstrated to measure small (µm-scale) displacements wirelessly in a wide (mm-scale) dynamic range at laboratory environment. The structure was shown to be highly suitable for being employed as a wireless and passive probe for detecting relative displacement and average strain in SHM. Subsequently, the comb-like NSRR architecture was exploited in measuring both the elastic and plastic deformation region displacement and strain of the standard steel rebar used in reinforced concrete members [41]. Finally, the disruptive effects of the complex electromagnetic media created by the combinations of concrete and rebar grids on the sensing were systematically studied, and solutions were proposed to overcome or to mitigate these challenges [42]. These preliminary measurements and additional tests performed in the presence of concrete and rebar grid proved that the sensing system comprising the modified comb-like NSRR probe together with an external antenna, which was optimized for better illumination and interrogation of the sensor, was effective.

In this paper, we introduce the displacement and strain sensing system finalized for use in the real-life scenario of being embedded inside the concrete. We present the results of the simply supported beam experiment with an actual scale beam sample where the NSRR probes are installed on the bottom longitudinal steel bars to detect the axial deformation of the bar as the beam bends due to the point load applied to the center of the beam. Comparing with data of the strain gages placed on the same steel bar, here it is shown that the system can accurately record the strain of the elements of the rebar grid in the elastic deformation region measurements. In addition, it is shown that the sensing system can also operate in the plastic deformation region, which is the case that can be encountered after a significant overloading such as an earthquake. We describe the calibration method for correctly transforming the measured frequency shift of the sensing system into strain. To the best of our knowledge, this is the first account of such a real-life experiment for a wireless and passive sensor utilized for SHM. It is also the first study that demonstrates a completely functioning RF displacement/strain sensor that is resistant to the disrupting effects of the complex electromagnetic medium constituted by the concrete and rebar grid. Hence, the practical challenges encountered during all phases of the experimental process are discussed, and solutions to these problems are shown throughout the manuscript.

## 2. Methods

### 2.1. Metamaterial-Based Sensing System

**Figure 1:**
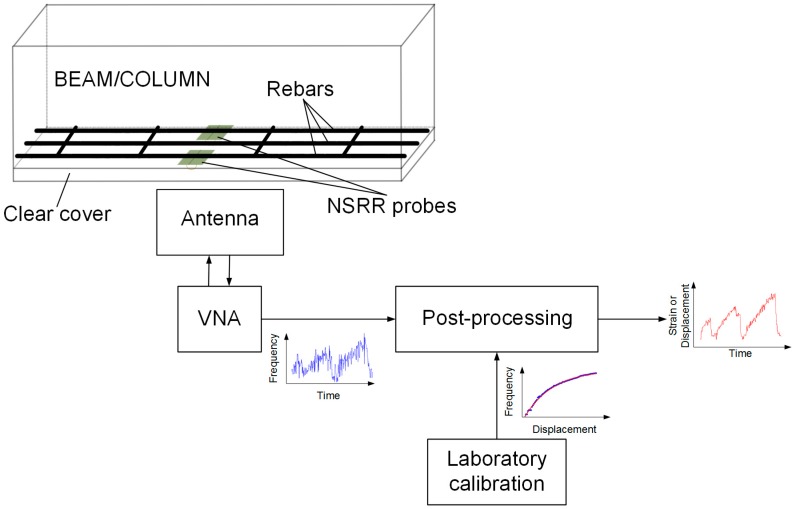
A diagram illustrating the working principle of the sensing system.

A block diagram illustrating the working principle of our sensing system is presented in Figure 1. The wireless sensing system comprises a sensing probe that is attached on the rebars inside a reinforced concrete member such as a beam or column and an external interrogating antenna. The antenna transmits the RF signal it is fed with from a vector network analyzer (VNA), and collects the backscattered signal. The signal is then post-processed using the calibration curve obtained in laboratory conditions, and basic signal processing, such as filtering or curve fitting. Each of these phases is explored in detail now. The sensing probe employed in the system is in comb-like NSRR geometry. Instead of the original single-piece structure that we previously proposed [39], here the probe is split symmetrically such that it has two freely-moving parts following our modified design [40]. These two parts are attached on two points on the rebar and the system monitors the separation between the attachment points. This makes the structure operate more like a displacement sensor than a strain meter, although the average strain can still be acquired by dividing the measured relative displacement by the original separation. The reasoning behind the splitting of the structure is to be able to capture larger displacements by removing the mechanical limitations dictated by the inelastic sensor substrate made of a dielectric material. The electrical connection between the two moving parts is made through a bonded 0.1 mm-radius wire. This NSRR architecture basically works as an LC resonator of which capacitance and inductance are determined by the number, length, and width of parallel comb-tooth pairs facing each other as well as the length of the connecting wire used. In operation, a change in the gap between these comb-tooth pairs affects the overall capacitance of the structure and leads to a shift in the resonance frequency. The sensor and the interrogating antenna have an electromagnetic interaction strengthened by near-field coupling. By this coupling, the sensor resonance frequency can directly be observed in the input impedance of the antenna by a spectrometer, e.g., a VNA as mentioned before. Owing to the 1-1 mapping between the measured resonance frequency and the separating gap between the comb-like NSRR parts, one can retrieve the displacement information from the telemetric measurements. Further detailed information on the sensor architecture including its resolution, sensitivity, linearity, dynamic range and monitoring distance can be found in [40,41]. These properties are summarized in Table 1.

**Figure 2:**
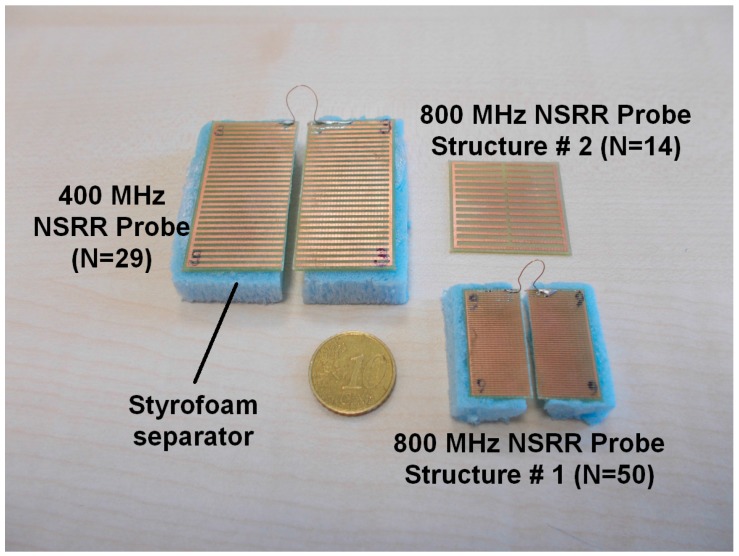
Displacement sensors designed in comb-like nested split-ring resonator (NSRR) geometry. Three different structures are shown. These vary in size and operating frequency. *N* is the number of the nested split-rings.

In practice, the NSRR probe is placed on a rebar in a concrete core, where a layer of concrete is present behind the sensor, along with a concrete or plaster clear cover in front of the sensor. Materials of concrete and steel as well as periodic placement of the longitudinal and vertical rebars and stirrups within the concrete generate different transmission and reflection properties and introduce disruptive electromagnetic effects on the operation of the sensing system. The effect of the complex electromagnetic media constituted by different combinations of these materials have been experimentally studied systematically by our group and was reported in [42]. This study showed that the most disruptive scenario was observed when a reinforced concrete block was placed immediately behind the NSRR probe. When the distance between the NSRR probe and the block was greater than 1.5 cm, this effect was negligible. However, for shorter distances, sensing became increasingly difficult and eventually unobservable. In order to cancel this effect, the NSRR probe was backed with a 1 cm thick Styrofoam separator (see Figure 2). Styrofoam was preferred due to its air-like electromagnetic properties. Other dielectric materials (such as cardboard or wood) were observed to have the effect of increasing the overall capacitance of the NSRR probe and resulting in a decrease in the resonance frequency; hence, were not selected. Three different designs are shown in Figure 2 for the comb-like NSRR sensor in different sizes and operating frequencies, where *N* is the number of the nested split-rings. The final form of the sensing system and experiment setup is explained in detail in the next section.

### 2.2. Experiment Setup

**Figure 3:**
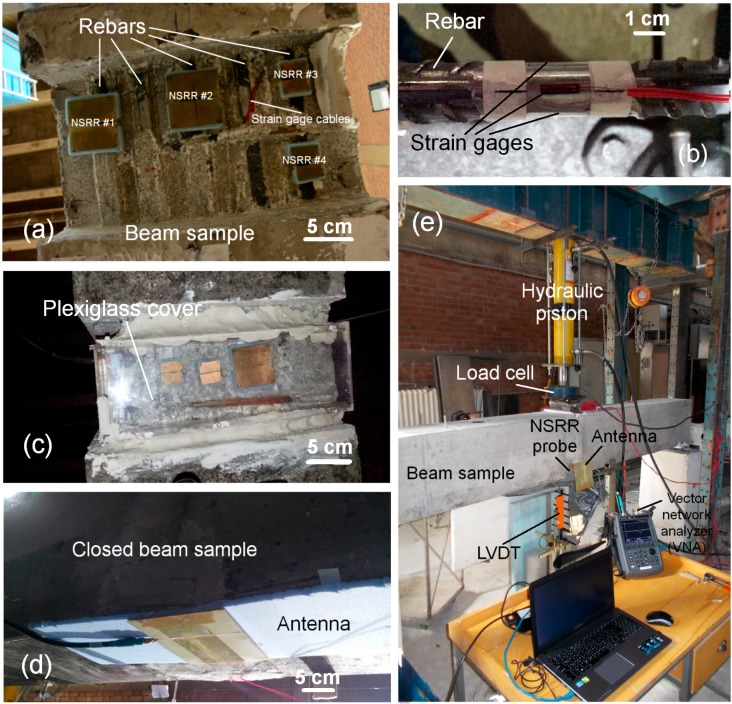
(**a**) The arrangement of the NSRR sensors actually used in the experiment at the bottom of the beam; (**b**) Close-up of a strain gage attached on a rebar before the concrete beam is produced; (**c**) Placement of the protective Plexiglas cover in front of the NSRR sensors (not the actual setup used in the experiment, but from a beam sample used for the preliminary tests before the actual experiment); (**d**) Placement of the antenna at the bottom of the beam, which is closed with a plaster layer after the placement of the sensors and the Plexiglas cover; (**e**) Simply supported beam experiment setup, where the slot antenna and a representative NSRR probe is shown at one side of the beam (the preliminary test setup).

In this study, real-life performance of the sensing system was investigated in a full-scale setup (Figure 3) where the NSRR probe was integrated into a beam sample, completely surrounded by concrete. Simply-supported beam experiments were conducted on this beam with the dimensions of 300 mm × 400 mm × 2950 mm (width, depth, length). The beam was placed horizontally on two fulcrums at each side along the length of the beam and mechanical force was applied by hydraulic pistons from the middle section downwards so as to bend the reinforced concrete beam and create an axial strain on the bottom longitudinal bars (see Figure 3e). The rebar grid inside the beam sample incorporated five 16-mm diameter deformed bars as well as 8-mm rectangular stirrups placed with a 20 cm separation. For the integration of the sensors, an 8-cm wide and 5-cm deep cavity was created at the bottom of the beam by using a Styrofoam block as a formwork before the concrete was poured. The Styrofoam formwork and concrete were rasped to reach the surface of the rebar, followed by the attachment of the two-part comb-like NSRR sensors on the rebars. As discussed earlier, a 1-cm thick Styrofoam spacer was used as a separator for eliminating the disruptive effect of the reinforced concrete behind the NSRR probe on the electromagnetic coupling. Each of the two parts of the separator was fixed on the rebar using a silicone paste through small cylindrical plastic pieces in order to have a point attachment. This way, the effect of strain propagation from rebar to the NSRR was minimized and the dynamic range of the sensor was significantly increased. Moreover, this approach confirms that the average strain information is accurate since the engineering strain can be calculated from the displacement measurement by dividing the data to the initial distance between the two attachment points and a fixing point as small as possible means increased accuracy. With the addition of Styrofoam layer, plastic pieces and rasping of the periphery of the rebars, the distance between the back of the NSRR probe and the concrete surrounding the rebar was measured to be around 1.2 cm.

A set of four NSRR probes was employed in the experiment, they were either one of the following two designs: The first design had an original resonance frequency (which is the resonance frequency when the edge-to-edge separation distance between the two NSRR parts, *d*, is equal to 0 and the length of the connecting wire is nominally set to 3.5 cm) at about 400 MHz, had a bigger footprint (4.7 cm × 4.7 cm) and incorporated 29 metal ring lines each of which had a thickness of 0.8 mm. The other one had an original resonance frequency at 800 MHz, had a smaller footprint (2.5 cm × 2.5 cm) and incorporated 50 metal rings with a thickness of 0.2 mm. As pointed out, the original frequency (*f*_0_) of a comb-like NSRR sensor, which occurs when there is no strain on the rebar, can be fine-tuned by the starting edge-to-edge distance (*d*) set between the two NSRR parts. In the experiments, on the first and the third of the five rebars at the bottom of the beam, NSRR #1 and NSRR #2, (which were in bigger footprint geometry) were attached, respectively. Two small-size sensors were located side by side on the fifth rebar, named as NSRR #3 and NSRR #4. No sensor was connected to the second and fourth rebars in order to prevent the intercoupling between the sensors. The arrangement of the sensors at the bottom of the beam is shown in Figure 3a. After the attachment of the NSRR probes, a concrete-gypsum mix based plaster layer was placed to close the gap of the beam. To protect the sensors from this plaster, a Plexiglas cover was used in front of the sensors as seen in Figure 3c. The final form of the closed beam is presented in Figure 3d. For the comparison of the data measured by the sensing system with an accurate reference, Kyowa KFG-5-120-C1-11L5M2R strain gages were employed (see Figure 3b). At each rebar, three gages were placed with 90° separation. The strain on a rebar was measured by an NSRR probe at one face, two strain gages at each side, and another opposing one, which was across the rebar. The VNA Agilent FieldFox N9915A as used. Vishay 5100B Scanner was used as the data logger.

The 400 MHz and the 800 MHz probes were monitored by two different microstrip single slot antennas designed at these frequencies. The bandwidth of the 800 MHz system is larger compared to the 400 MHz system, while the latter has an interrogation distance advantage over the former. Since the antenna acts as the transceiver in the single channel communication between the NSRR probe and itself, only one of these systems can be employed for monitoring strain at the same time with one network analyzer. On the other hand, the systems do not affect each other and undisturbed responses can be obtained from each sensor at different measurements.

During the preliminary tests, two criteria have been identified as being very critical in terms of the success of the experiment and the accuracy of the results. These points are given as follows:

(1) The concrete has to be rasped until the rebars are visible and the sensors have to be attached directly on the rebars instead of on the concrete. This is vital since the propagation of strain from the rebars onto the concrete is experimentally observed to be hindered by formation of cracks on the concrete. This results in a failure to correctly capture the elongation or contraction. When the sensors are placed directly on the rebars, sensing becomes possible.
(2) The antenna has to be kept at a constant distance from the sensors (normal to the sensor surface, *i.e.*, *z*-direction), with respect to the moving beam sample. The reason is that the disruptive effect of the relative positions of the antenna and the sensors on the electromagnetic coupling is amplified by the presence of the concrete. The effect of the relative positions of the antenna and the sensors is observed to be minimal at a large dynamic range (such as thousands of microstrains or a few millimeters of displacement), but becomes very critical in the elastic deformation region of steel where the maximum displacement is in the order of a few tens of micrometers. Thus, the correct location of the antenna should be determined, which can be done by moving the antenna in the *x*-*y* plane until the same resonance frequency peaks, obtained in laboratory conditions, are observed again on the beam setup. When the location of the antenna is determined, a transformation curve produced by laboratory experiments can be applied directly to convert the frequency shift into displacement and strain. In our experiments, steady positioning of the antenna is achieved by fastening it on the beam through a Styrofoam layer as shown in Figure 3d.

These two criteria have been taken into attention at all experiments on the actual beam sample for obtaining the results given in the next section.

## 3. Results and Discussion

### 3.1. Elastic Deformation Region of Steel

**Figure 4:**
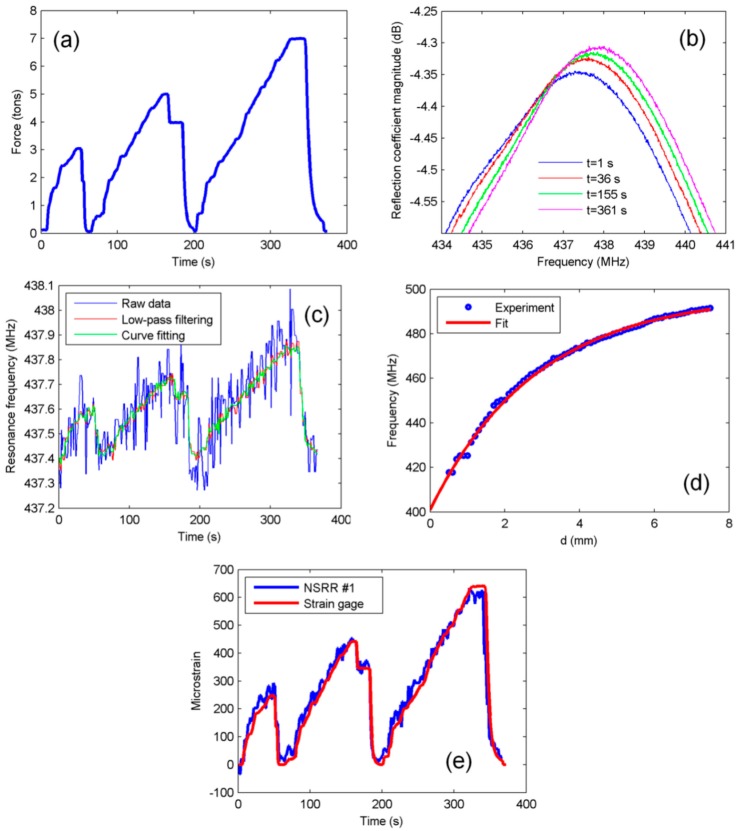
Elastic deformation region experiment results of NSRR #1: (**a**) applied vertical force read from the load cell; (**b**) NSRR #1 resonance frequency peaks with strain forming due to applied load at several time instants; (**c**) NSRR #1 peak frequency plotted by using the raw reflection coefficient data (**blue**), after a low-pass filter is applied to the raw data (**red**), and after 5th degree polynomial fitting is applied to the raw data (**green**); (**d**) a typical transformation curve (in air medium) obtained before the beam experiments by a controlled translational stage, which is used for converting the frequency shift into displacement and strain; and (**e**) strain obtained by the NSRR #1 plotted *versus* time, and compared to the data from the strain gages.

On the beam sample, several load-and-release experiments were performed within the elastic deformation region of the steel rebars. The variation of the vertical force applied to the beam and monitored via the load cell in one of the elastic-region experiments is given in Figure 4a. As seen in the figure, the force was first increased slowly to 3 tons with the manual hydraulic piston and then the piston was released so that the system has a net force equal to 0 in the first 45 s. It was then increased to 5 tons and again decreased to 0. Finally, it was increased to 7 tons and decreased to 0 once again. The reasoning behind the incremental loading to different force levels is to test how well the sensing system can follow the force-deformation regime, and to observe its response to different force levels. As a result of the loading and releasing, the separation between the NSRR probe parts is increased and decreased, respectively, leading to first a reduction and then an increment in the overall capacitance of the sensor. Since the resonance frequency is inversely proportional to the capacitance, we expect the frequency shift with time to follow the strain gage results, and to more or less imitate the force regime in the elastic range. The resonance frequency manifests itself as a local peak in the system impedance monitored through the antenna. Therefore, the movement of this peak should match the behavior measured form the strain gages. The shift of the frequency peak of NSRR #1 can be observed via the system reflection coefficient plots recorded during the experiment at the first second and at time instants corresponding to the 3, 5 and 7 tons of force, as shown in Figure 4b.

When the NSRR #1 peak frequencies of the raw data corresponding to every time instant are plotted, the blue curve shown in Figure 4c is obtained. Although the level of noise is high for the blue curve obtained from the raw data, it is still distinguishable that the shape of the frequency shift is quite similar to the one obtained from the strain gages. Several numerical methods can be implemented to eliminate the noise and obtain a better curve. Among these, two have been given special attention. First method is to design and apply a low-pass filter to discard the high frequency components in the waveform. With this goal, the Fast Fourier Transform (FFT) of each curve was taken and a proper cut-off frequency of the filter was determined by inspection of the spectrum. Then, a 100th order FIR filter was designed and was applied to the signals. Maxima of each curve were used to plot the frequency shift. The resulting waveform was plotted with red in Figure 4c. The second method was based on curve-fitting. A 5th degree polynomial fit was applied to a selected region around the peak of each reflection coefficient curve, and the maxima of each fit were found afterwards. The polynomial degree, the number of data points and the width of the region were all selected heuristically. The result is plotted with green in Figure 4c. When all three curves are compared, it is clear that both methods help to dispose of the noise significantly, but neither the curve resulting from the low-pass filtering nor curve fitting has a distinct advantage over the other. This implies that both methods can be utilized for post processing the frequency shift data. For the sake of maintaining consistency, we choose to proceed with filtering for all plots in the document.

As shown in Figure 1, in order to convert the measured frequency shift data into displacement and strain, a preformed transformation curve is necessary. This calibration curve, which defines the relationship between the frequency shift and the displacement, strongly depends on the properties of the complex electromagnetic medium formed by the concrete and metal rebar grid as previously shown by our group [42]. Therefore, two different calibrations can be defined for two limiting cases: (1) when the surrounding medium is air, *i.e.*, it is assumed that the sensing system is completely free of the disrupting effect of the concrete and the rebar grid; and (2) when the 1 cm thick foam is placed between a reinforced concrete block and the NSRR probe. The situation in practice is neither of these two cases but something in between since the distance of the back of the NSRR probe to the reinforced concrete is actually more than 1 cm because of the rasping and the presence of cylindrical plastic pieces. An experimental curve obtained by a controlled translation stage when the medium is air, is given in Figure 4d along with the exponential fit. The slope of this curve is decreased due to the effect of concrete [42]. Here, the curve utilized for the transformation of the resonance frequency shift into the displacement change takes into account the effect of the reinforced concrete and is described as $f\left(d\right)=410+75\left(1-e^{-0.35d}\right)$. This fit is derived by a controlled laboratory experiment where the actual experiment setup is mimicked as close as possible. In this experiment, the distance between the NSRR probe and the concrete block was modified and the change of fit parameters was observed. Here, the fit for the case when the distance between the NSRR probe and the concrete is taken as 1.2 cm is employed. Inverse of this fitted curve is used to convert the measured frequency shift into the displacement. Strain is then calculated by dividing the displacement to the distance between the attachment points of the two parts of the NSRR, which is 26 mm for NSRR #1.

Strain data obtained from NSRR #1 and from the strain gages are plotted *versus* time in Figure 4e. As mentioned before, at each rebar, three strain gages were placed at the same location with the midpoint of the NSRR probe such that each element makes a 90° spacing with its neighbor. As the beam bends due to the applied vertical force, the bottom longitudinal rebars not only elongate but also bend. Bending of the rebar causes the displacements at the top and the bottom of the rebar to be different from each other. In order to cancel the effect of the bending of the rebar and to calculate the axial deformation of the bar only, the sum of the strains of the opposing elements (*i.e.*, the elements across each other) are assumed to be the same. The data for the strain gages, which are compared to the data from the NSRR sensor, are actually the result of the subtraction of the strain of the gage across the NSRR probe from the sum of the strains from the two neighboring gages. The strong matching shows that the system can correctly track the elongation and the contraction of the rebar with a micrometer-level sensitivity, and employing the translation stage calibration yields accurate results.

**Figure 5:**
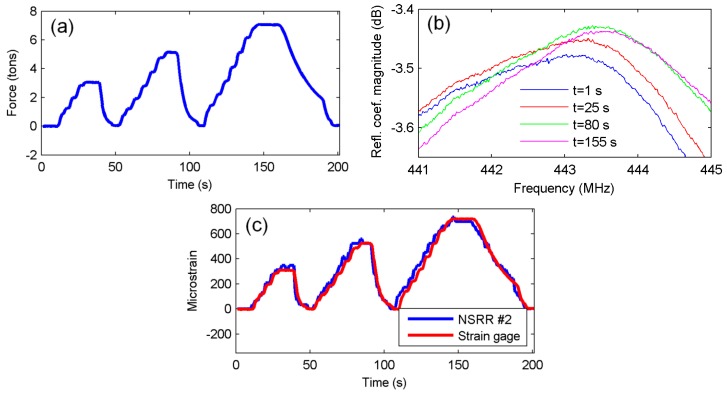
Elastic deformation region experiment results of NSRR #2: (**a**) applied vertical force read from the load cell; (**b**) NSRR #2 resonance frequency peaks with strain forming due to applied load at several time instants; and (**c**) strain obtained from NSRR #2 plotted *versus* time, and compared to the data from the strain gages.

**Figure 6:**
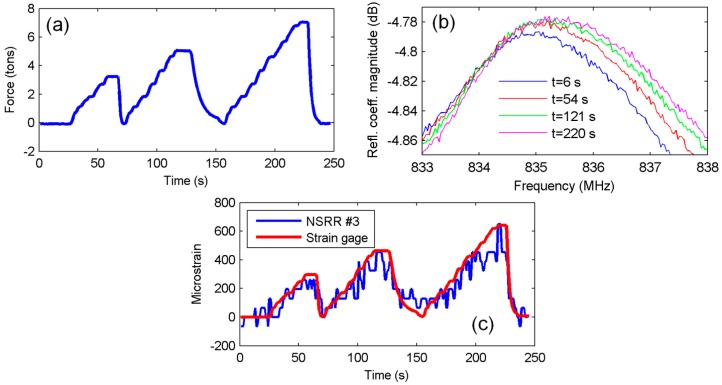
Elastic deformation region experiment results of NSRR #3: (**a**) applied vertical force read from the load cell; (**b**) NSRR #3 resonance frequency peaks with strain forming due to applied load at several time instants; and (**c**) strain obtained from NSRR #3 plotted *versus* time, and compared to the data from the strain gages.

Elastic deformation experiments were repeated and data from other NSRR sensors were also recorded. The data for NSRR #2 and NSRR #3 are shown in Figure 5 and Figure 6, respectively. The distance between the attachment points of NSRR #2 is 30 mm. For NSRR #3, which has a smaller footprint geometry, this value is 10 mm. For NSRR #4, no proper data could be recorded due to bad attachment of the probe. The strain obtained by NSRR #2 is observed to agree with the strain gage data closely. On the other hand, for NSRR #3, the data are much noisier compared to the results from the bigger footprint probes. This can be attributed to poor coupling between the probe and the antenna, and more dominating effect of the clutter in higher frequency.

In previous studies, the sensing system was shown to acquire accurate data at tensile tests where a single rebar with a diameter of 8 mm was elongated under the effect of a vertical force [41]. In the simply supported beam experiments, a beam undergoes bending deformation where the transverse loading causes compressive strains at the top and tensile strains on rebars at the bottom. Despite the presence of concrete surrounding the rebar, both experiments are observed to yield similar results. In the elastic region, where the induced deformation is reversible, the maximum level of strain is around 2000 microstains. Thus, this value set a maximum limit for the experiments, where the maximum strain level attained was around 800–900 microstrains. In the elastic measurements, it is important that the sensing system can record accurate data at such small strain levels, especially when the following challenges are considered: (1) the fact that the sensor moves up and down with the vertical force applied on the beam (mechanical changes generally result in deviations in the data, especially in elastic region); and (2) the environment measurement is a complex electromagnetic medium. The elastic deformation behavior of the beam is observed through application of a cyclic force regime. Within the elastic deformation range, the relationships between every parameter (force, stress, strain, relative displacement, *etc.*) are linear. These linear relationships can clearly be seen in Figure 4, Figure 5 and Figure 6, where it is observed that the ratio of the force to the strain stays approximately the same during the whole experiment.

### 3.2. Discrete-Time Measurements

**Figure 7:**
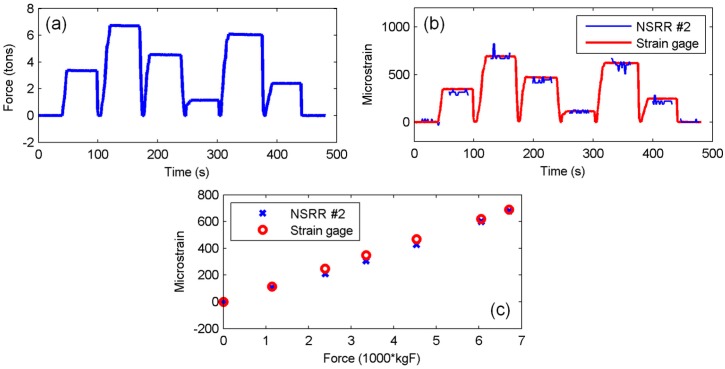
Discrete-time measurements: (**a**) applied force levels; (**b**) strain read by the sensing system with NSRR #2 and by strain gages, plotted *versus* time; and (**c**) average of the strain values for each force level, plotted *versus* the force.

In addition to dynamic measurements where the sensing system collects data continuously for a period of time, another real-life application is to take measurements at different time instants, e.g., for periodic inspection. Experiments were also carried out for testing the discrete time measurement performance of the sensing system. For this purpose, eight different levels of force were applied on the beam, the first and the last of which being 0 tons as shown in Figure 7a. The sensing system recorded measurements for a period of time at each force level. The values of strain forming at each force level are shown in Figure 7b for both NSRR #2 and the strain gage. In Figure 7c, the random force and strain values are sorted and average microstrain value is plotted *versus* applied force. It can be observed in the figure that the agreement between the strain gage and sensor data is once again satisfactory. In discrete-time measurements, averaging of the data is important in reducing the system noise level that can be increased due to any electrical or mechanical change in the surrounding environment. The resolution of the sensing system, which can be defined as the minimum measured quantity, is calculated to be on the order of microstrains for both discrete and continuous time elastic deformation region measurements. The sensitivity of the system can be defined as the frequency shift change per unit strain or displacement. In the discrete-time sensing experiment, the frequency shift from 0-strain case to the maximum (690 microstrains) strain case is measured as 460 kHz. Since the distance between the attachment points of NSRR #2 is 30 mm, the relative displacement between the two points is 20 micrometers, which yields a sensitivity of 22 MHz/mm. Distinguishing kHz level peak shifts is possible via the network analyzer, and hence, we can obtain a resolution in microstrain level. These values can be considered as fairly sufficient for SHM of reinforced concrete members.

### 3.3. Plastic Deformation Region of Steel

An important property brought in by the sensing system is to monitor displacement and strain at the plastic deformation region of steel, where traditionally used devices such as strain gages fail to capture due to their limited dynamic range. The measurement limit of the strain gages is closely related with the way they are installed on rebars. Generally, strong adhesives are used for this purpose, but such an installation approach only enables elastic region measurements (strains up to 0.2%–0.3%), after which the strain gages tear down since the strain forming on the rebar causes the adhesives to rip off. Special measures must be taken for high elongation measurement (fracture strain of rebar, *i.e.*, ~20%–30%) but they require practical challenges such as curing at elevated temperatures for several hours, special protective coating, and special surface preparation procedures. These severe installation demands are very difficult to apply in a real-life application. On the other hand, it was demonstrated that our system could successfully measure strains until the fracture of the rebar in a steel rebar tensile test experiment [41].

**Figure 8:**
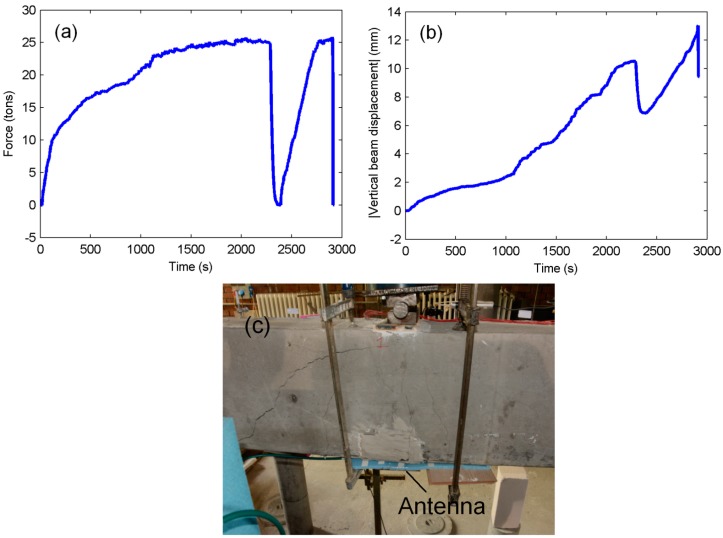
Plastic deformation region measurements: (**a**) applied vertical force read from the load cell; (**b**) absolute vertical displacement of the midpoint at the bottom of the beam, LVDT reading; and (**c**) the beam sample with visible cracks after the experiments.

The loading regime during the plastic deformation experiment is shown in Figure 8a. At *t* = 2287 s, the force on the beam was decreased to the point of no loading, and it was again increased to 25 tons in a shorter period of time. Then, at *t* = 2900 s, loading was stopped before the beam failed. The absolute value of the displacement of the bottom midpoint of the beam sample was monitored by a Linear Variable Differential Transformer (LVDT), and the result is plotted in Figure 8b. It can be observed that the beam became subject to a maximum of 13 mm vertical displacement at the end of the experiment. This value is quite large when compared to the value of beam deflection in the elastic range experiments (which was only around 0.4 mm) when 7 tons of force were applied.

**Figure 9:**
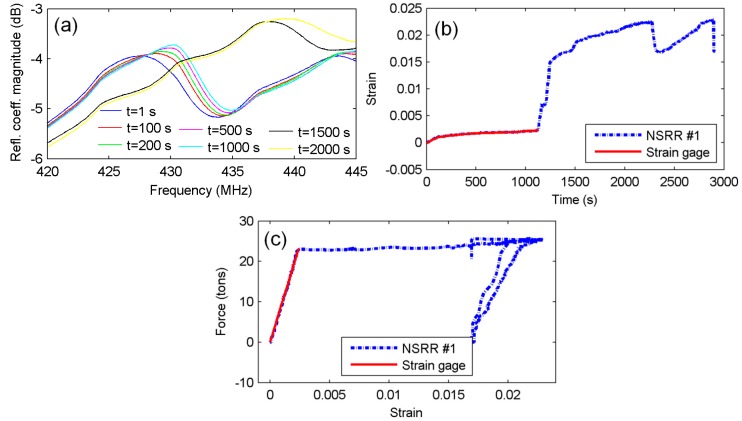
Plastic deformation region measurements: (**a**) NSRR #1 resonance peaks at several time instants; (**b**) strain obtained by NSRR #1, plotted *versus* time and compared to the data from the strain gages until the strain gages break down; and (**c**) the force–strain curve plotted with both NSRR #1 and strain gage data.

The sensing system recorded data at every 3 s during the experiment. The shift of the filtered frequency peaks corresponding to NSRR #1 is shown in Figure 9a through data plotted for several time instants. Especially, the big jump between *t* = 1000 s and *t* = 1500 s can clearly be observed. The strain data obtained from NSRR #1 is plotted in Figure 9b, and compared to the strain gage data. At *t* = 1128 s, corresponding to 2450 microstrains, an immense increase is observed in strain due to yielding of the rebar and flexural cracks in the concrete at the middle of the beam, which forced the strain gages beyond their limit, eventually causing them to break down. Therefore, after that instant, the only information source left regarding the strain forming on the rebars was our wireless sensing system. Based on the very good agreement between the two results in the elastic deformation region and rebar tensile test performance of the sensing system, it can be assumed that the measurement of the NSRR sensor after the breakdown of the strain gages is also acceptable. Furthermore, the force–strain curve shown in Figure 9c is consistent with the expectations, thus we can deduce that the readings are accurate. A maximum strain of 0.023 is measured at the instant of the highest force, which means that 0.6 mm displacement occurs on the rebar between the two attachment points. The dynamic range of the system was previously shown to be over 20 mm in [40], which implies that the system practically does not possess any limits regarding the maximum measurable strain or displacement. Hence, it is demonstrated that the system is sufficient for monitoring the reinforced concrete members until failure.

Due to the presence of concrete holding the rebar grid, the curve obtained in Figure 9c is slightly different looking than a classic stress-strain curve of a steel rebar. The reason for this is that the concrete hinders reaching higher levels of force and strain where the beam fails. In Figure 9c, the elastic and plastic regions are clearly separated. However, since the level of the applied force cannot be increased to even higher values (unlike the tensile loading experiment) due to failure of the beam, a maximum of 23,000 microstains is measured on the rebar where NSRR #1 is attached. This prevents the initiation of the strain hardening region, which was observed to start when the applied force was increased past over 30,000 microstrains in the previous tensile tests [41]. Therefore, at the instant when the plastic deformation experiment was terminated, the rebars were at the yielding phase, where the level of the applied force stays more or less the same while the strain induced on the rebars is increased. This can also be observed in Figure 8a, where the level of applied force does not vary very much; and also in Figure 9c, where the change of force is minimal, while the strain increases from 2000 microstrains to over 20,000 microstrains.

**Figure 10:**
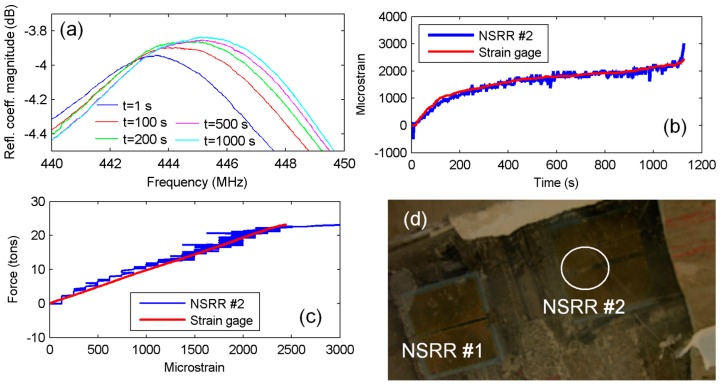
Plastic deformation region measurements: (**a**) NSRR #1 resonance peaks at several time instants; (**b**) strain obtained by NSRR #1 is plotted *versus* time and is compared with the data from the strain gages until the strain gages break down; (**c**) the force–strain curve plotted with both NSRR #1 and strain gage data; and (**d**) the piece of concrete stuck in NSRR #2 during the experiment.

The results of NSRR #2 were also recorded during the experiment. However, due to a physical effect, an artificial resonance frequency change was observed during the yielding. Therefore, only the results for the first 1128 s are shown for NSRR #2 in Figure 10. When the plaster layer and the Plexiglas protection cover were removed after the experiment, it was observed that a piece of concrete had come off and had been stuck between the two NSRR parts, leading to a big change in the capacitance, as shown in Figure 10d. It can be observed in Figure 10 that the system operated thoroughly until this incident. Strong agreement between the NSRR probe and strain gage results prove that the laboratory calibration is also successful in transforming the frequency shift into strain.

## 4. Conclusions

In this study, a passive and wireless sensing system was employed in a simply supported beam experiment for detection of displacement and strain occurring on the rebars. The system utilizes the electromagnetic coupling between a transceiver antenna and several nested split-ring resonator (NSRR) probes, which are metamaterial-inspired, high-sensitivity structures. Each probe consists of two parts that are fastened on the rebar surface with point attachments, and the overall capacitance of the probe changes with a variation in the displacement between these attachment points. A Plexiglas protection cover was placed between the sensors and the plaster layer, which encloses the beam completely. A premade calibration curve created with the help of the laboratory experiments that mimicked the complex electromagnetic medium of concrete and rebars was used to transform the measured resonance frequency shift into displacement and strain. Bending tests were repeated for several probes in the elastic deformation region of the rebars and the results were shown to agree well with those of the strain gages. Then, the system was tested in the plastic deformation region, where a vertical force of up to 25 tons was applied, creating a mid-span displacement of 13 mm in the beam. Data were recorded continuously in both elastic and plastic regions in a real-life scenario, and the readings of the sensor were shown to match with those of the strain gages through correct calibration. It was also demonstrated that the system could be used to record data at discrete times instead of continuous monitoring, and that the same calibration was also valid for this case. The results of the experiments prove that the system exhibits a high sensitivity and resolution (μm and microstrain level) along with a wide dynamic range (mm), unlike strain gages and other wireless passive sensors in literature. To the best of our knowledge, there is no work in the literature that demonstrates a real-life performance of a wireless and passive sensor integrated inside a reinforced concrete member. The sensing system is shown to be a strong candidate for wireless SHM as well as for the measurement of strain and displacement after a significant overloading such as an earthquake.

## Figures and Tables

**Table 1:** Sensing system properties for the measurements carried out in air and the complex medium consisting of the concrete, the rebar grid, and the concrete cover [40,42].

	Air	Complex Medium
Sensitivity	12.7 MHz/mm (for 2 mm range)	7.5 MHz/mm for the worst case
Resolution	~0.5 µm	~1 µm
Dynamic range	>20 mm	>20 mm
Linearity (*R*^2^)	>0.99 for 3–8 mm	>0.99 for 3–8 mm
Monitoring distance (*D_m_*)	<60 cm	<60 cm

## Abbreviations

The following abbreviations are used in this manuscript:

SHM	Structural health monitoring
NSRR	Nested split-ring resonator
RFID	Radio-frequency identification
LVDT	Linear variable differential transformer
VNA	Vector network analyzer
